# The optimal regional irradiation volume for breast cancer patients: A comprehensive systematic review and network meta-analysis of published studies

**DOI:** 10.3389/fonc.2023.1081201

**Published:** 2023-01-31

**Authors:** Wei-Xiang Qi, Lu Cao, Cheng Xu, Gang Cai, Jiayi Chen

**Affiliations:** Department of Radiation Oncology, Ruijin Hospital, Shanghai Jiaotong University School of Medicine, Shanghai, China

**Keywords:** regional irradiation volume, breast cancer, network meta-analysis, survival, systematic review

## Abstract

**Background:**

Currently, the optimal adjuvant regional nodal irradiation (RNI) volume for breast cancer (BC) remained controversial. We aimed to define the optimal RNI treatment volume for BC by using a comprehensive network meta-analysis (NMA) of published studies.

**Materials and methods:**

PubMed, Embase, Medline, and Cochrane Central Register of Controlled Trials were searched from database inception to 30 May 2022. Studies assessing different adjuvant RNI volumes for BC were eligible for inclusion. The primary outcome was overall survival (OS), and secondary outcome was disease-free survival (DFS) and distant-metastasis-free survival (DMFS).

**Results:**

A total of 29,640 BC patients from twenty studies were included. The pooled hazard ratio demonstrated that internal mammary node irradiation (IMNI) in BC patients significantly improved OS giving HR (hazard ratio) of 0.87 (95%CI: 0.83–0.91, *p*<0.001), DFS with HR of 0.78 (95%CI: 0.68–0.90, *p*<0.01), and DMFS with HR of 0.87 (95%CI: 0.79–0.97, *p*<0.01) when compared to controls. Sub-group analysis indicated that RNI with IMNI significantly improved OS (HR 0.87, 95%CI: 0.81–0.93, *p*<0.01), DFS (HR 0.65, 95%CI: 0.56–0.77, *p*<0.01), and DMFS (HR 0.90, 95%CI: 0.82–0.98, *p*=0.02) when compared to RNI without IMNI. NMA showed that CW/WB (chest wall/whole breast) + RNI with IMNI significantly improved DFS (HR 0.93, 95%CI: 0.86–1.00) and DMFS (HR 0.90, 95%CI: 0.81–0.99), but not for OS (HR 0.93, 95%CI: 0.84–1.03) when compared to CW/WB alone. Based on the analysis of the treatment ranking, CW/WB+RNI with IMNI appeared as the best treatment approach for BC patients.

**Conclusions:**

Our pooled results demonstrated that RNI with IMNI yielded a significant survival advantage for BC patients. NMA showed that CW/WB+RNI with IMNI was the optimal radiation volume for BC patients.

## Introduction

According to the global cancer statistics 2020, female breast cancer (BC) ranked the most commonly diagnosed cancer, accounting for 11.7% of total tumors with an estimated 2.3 million new cases (1). Radiation therapy (RT) played a key role in the management of BC after breast-conserving surgery or mastectomy, and regional nodal irradiation (RNI) was widely used for the treatment of node-positive BC due to its potential survival benefit (2). Since the publication of two large phase III randomized controlled trials MA-20 (3) and EORTC-22922/10925 (4), comprehensive RNI including supraclavicular lymph nodes (SVCs) and internal mammary lymph nodes (IMNs) had become the standardized adjuvant RT therapy for high-risk node-negative or involving one to three node-positive BC in international guidelines. However, despite an abundance of data supporting the benefit of comprehensive RNI for BC, uncertainty existed regarding the appropriate RNI volume for BC. Indeed, significant inter-physician variability existed in real-world practice especially in IMN irradiation (IMNI) because the inclusion of the IMN would lead to cardiopulmonary extra doses, which increase the risks for developing late adverse events such as cardiac events and pulmonary fibrosis. In a more recent randomized clinical trial (KROG 0806), the authors failed to demonstrate superiority of the IMNI group over those treated without IMNI for node-positive BC patients because of sample size calculation issue, poor protocol compliance, and RT quality assurance issue (5). Thus, an unanswered question about the optimal regional nodal irradiation volume for BC patients remains, and the optimal RNI strategy gained great interest for radiation oncologists with the aim of archiving maximal clinical benefit of RNI, with the minimal possible radiotherapy toxicity to maintain the quality of life. As a result, we performed the present comprehensive meta-analysis to investigate the efficacy of radiotherapy with IMNI vs. RNI without IMNI in BC patients. In addition, we also compared the efficacy difference of RNI regimens head-to-head *via* network meta-analysis in terms of combined clinically meaningful overall survival (OS), disease-free survival (DFS), and distant-metastasis-free survival (DMFS) benefits.

## Materials and methods

### Data source

Several databases including PubMed, Embase, Medline, and Cochrane Central Register of Controlled Trials were searched for relevant studies. Studies comparing different regional nodal irradiation volumes were included. The search keywords were breast cancer, breast carcinoma, radiotherapy, regional node radiation, and clinical studies. Clinical studies should meet the following criteria (1): clinical studies involving BC patients (2), clinical studies comparing efficacy of different adjuvant RNI volumes, and (3) available survival data regarding RNI in BC patients. BC patients treated with neoadjuvant therapy were excluded for analysis in the present study.

### Data extraction

Four independent investigators conducted the data extraction, and any discrepancy between the reviewers was resolved by consensus. The following information was extracted for each study: first author’s name, year of publication, number of enrolled patients, study design, radiation regimen, main inclusion characteristics, radiotherapy dose, and median follow-up time. If the radiation volume of RNI was not specifically defined including RNI+SCN (supraclavicular lymph node) and/or RNI+IMNI and/or RNI+SCN+IMNI, we defined it as mixed RNI group. The primary outcome of interest was OS, and the secondary outcomes were DFS and DMFS.

### Outcome measures

The outcome data were pooled and reported as hazard ratio (HR). Between-study heterogeneity was estimated using the χ^2^-based Q statistics (6). Heterogeneity was considered statistically significant when *p*_heterogeneity_< 0.1. The presence of publication bias was evaluated by using the Begg and Egger tests (7, 8). A statistical test with a p-value <0.05 was considered significant. Sensitivity analysis was performed to assess the bias risk of one single study on the pooled result by a leave-one-out approach. Study quality of prospective randomized studies was assessed by using the Jadad scale based on the reporting of the studies’ methods and results (9). The quality of the retrospective or non-randomized studies was evaluated according to the Newcastle–Ottawa Scale (NOS) (10). For studies that did not report HR of OS, DFS, or DMFS, we used Engauge Digitizer 4.1 software to calculate the HR and 95% CI from the survival curve described by Tierney et al. (11).

An NMA offered methods to visualize and interpret a broader picture of current evidence and assessed the comparative effectiveness among various RNI volumes. Therefore, a network meta-analysis was performed using a frequentist framework (12). A network plot was generated for each disease setting to show all interventions included in the NMA. Comparative effectiveness results of all possible RNI comparisons were summarized with an HR and 95% CI (13). We investigated which RNI treatment volume most effectively reduced the hazards of BC progression, distant metastasis, and death by allowing multiple comparison treatment effects. Rank probabilities of treatments for efficacy were estimated by p-score. When the treatment chosen was the best option, the p-score approached 1 (100%), while p-score for the worst treatment option approached zero. Statistical analysis was performed using R software (Version 4.1.1, “meta,” “netmeta” package; R Foundation for Statistical Computing, Vienna, Austria) and Review Manager (RevMan) Version 5.0 (Copenhagen: The Nordic Cochrane Centre, The Cochrane Collaboration, 2008).

## Results

### Search results

**Figure 1:**
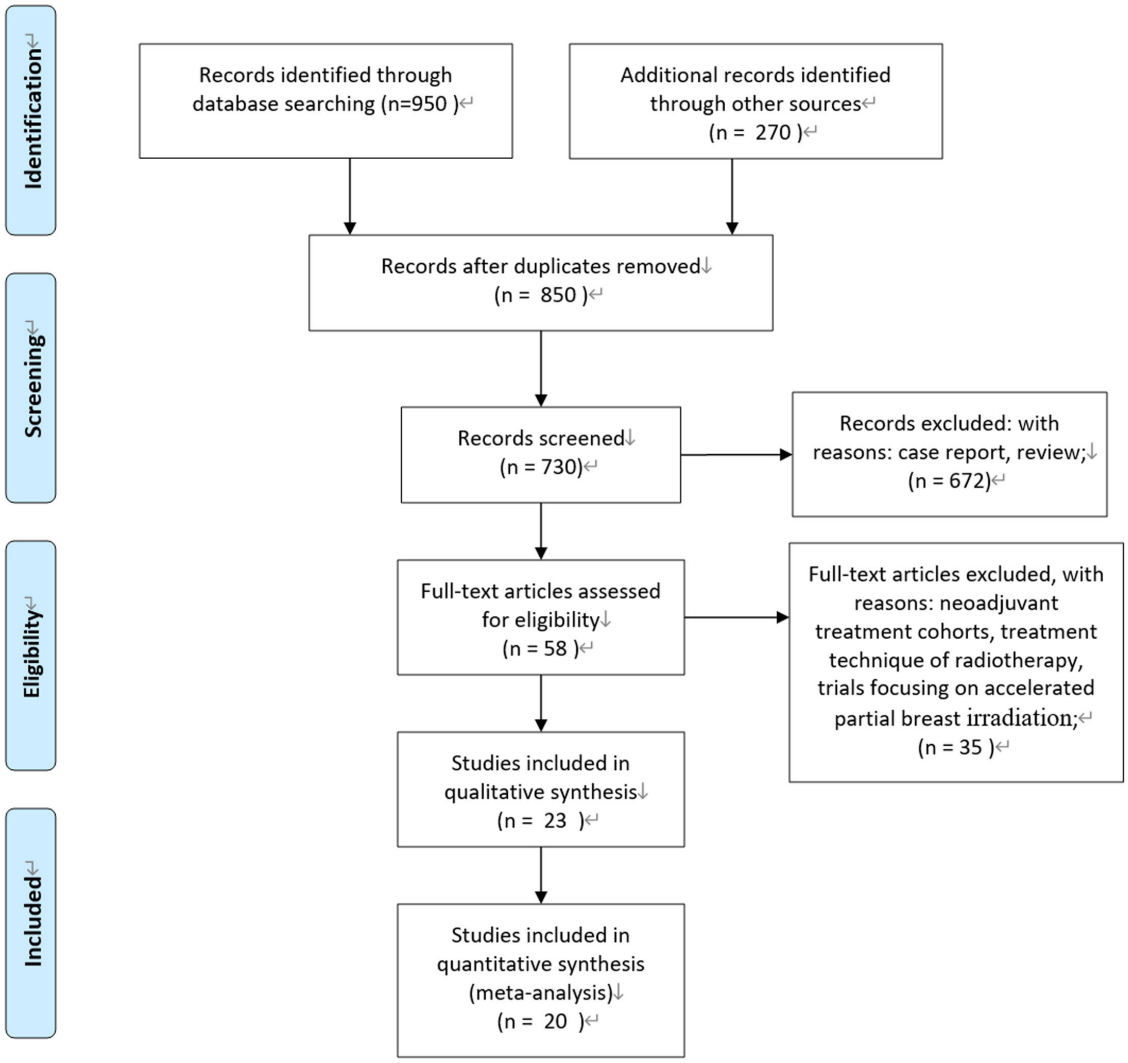
PRISMA flow diagram.

According to the Preferred Reporting Items for Systematic Reviews and Meta-Analysis (PRISMA) statement, we conducted the present meta-analysis (14). Our initial search yielded 850 potentially relevant reports. After excluding review articles, neoadjuvant treatment cohorts, treatment technique of radiotherapy, studies focusing on accelerated partial breast irradiation, case reports, meta-analyses, and observation studies, a total of 23 clinical studies were included. After reviewing the included studies, three studies were undated analysis of previously published studies (15–17). Finally, 20 publications of 23 clinical studies were included (3–5, 15–34). Of them, five studies were prospective randomized controlled trials. One study was a nationwide, prospective cohort study, and patients with right-sided disease were allocated to IMNI, whereas patients with left-sided disease were allocated to no IMNI because of the risk of radiation-induced heart disease (25); two studies were individual meta-analyses of two prospective cohorts (33, 34); and the remaining 12 studies were retrospective publications. **Figure 1** show the process of selection.

### Characteristics of the included studies

The main characteristics of included studies are summarized in **Table 1**. A total of twenty studies comprising 29,640 patients met the eligibility criteria. Both early-stage BC with pT1 or pT2 tumors with pN0 or pN1 disease or locally advanced BC patients were included. Patients treated with mastectomy or breast-conservation surgery (BCS) and planned axillary lymph node dissection were included. The median follow-up time ranges from 4.5 to 30 years. The common prescribed radiation dose for CW/WB and RNI was 45–50 Gy in conventional fraction size. The detailed information of the included studies is listed in **Table 1**.

**Table 1:** Baseline characteristics of 20 included trials.

Study/years	Study design	Recruitment period	RT regimen	No. of patients	Main inclusion characteristics	Radiotherapy dose	Median follow-up
KROG-0806/2022	Prospective randomized trial	2008-2020	WB/CW+SCN±AXL+IMN	362	N+	CW/WB: 45-50.4Gy/25-28Fx RNI: 45-50.4GY/25-28Fx	8.4 y
			WB/CW+SCN±AXL	373			
DBCG-IMN/2022	Prospective cohort trial	2003-2007	Right sided: WB/CW+SCN±AXL+IMN	1491	Early-stage N+	CW/WB: 48Gy/24Fx RNI: 48GY/24Fx	14.8y
			Left sided: WB/CW+SCN±AXL	1598			
Sit et al/2022	Retrospective study	2005-2014	WB/CW + mixed RNI	885	pT1-2N1M0 low risk (HR+, Grade 1-2, HER-2 negative)	NA	9.2y
			WB/CW	284			
DBCG 82bc trial/2022	Prospective randomized trial	1982-1990	CW+SCN+AXL+IMN	1538	Stage II-III	CW/WB: 48-50Gy/24-25Fx RNI: 48-50GY/24-25Fx	30y
			No RT	1545			
Cho W.K. et al/2021	Retrospective study	2006-2011	CW+SCN±AXL+IMN	105	N+	CW: 45-50.4Gy/25-28Fx RNI: 45-50.4GY/25-28Fx	7.9y
			CW+SCN±AXL	243			
French (Hennequin et al)/2013	Prospective randomized trial	1991-1997	WB/CW+SCN±AXL+IMN	672	N+ or medial/central tumor	50Gy or equivalent	8.6y
			WB/CW+SCN±AXL	662			
MA.20 (Whelan et al)/	Prospective randomized trial	2000-2007	WB+SCN±AXL+IMN	916	N+ or high risk N0 (tumor size≥5cm,LVI+,Grade 3 or ER-)	WB: 50Gy/25Fx RNI: 45GY/25Fx	9.5y
			WB	916			
EORTC 22922/10925 (Poortmans et al)	Prospective randomized trial	1996-2004	WB/CW+SCN±AXL+IMN	2002	N+ or medial/central tumor	CW/WB: 50Gy/25Fx RNI: 50GY/25Fx	15.7
			WB/CW	2002			
Wang X. et al/2020	Retrospective study	2007-2010	WB/CW+SCN±AXL+IMN	390	IBC without neoadjuvant treatment	CW/WB: 45-50Gy/25Fx RNI: 50GY/25Fx	8.17y
			WB/CW+SCN±AXL	482			
Park S.H. et al/2020	Retrospective study	2007-2016	WB + mixed RNI	36	T1-2N0-1M0 IBC	WB:45-60.4Gy; boost: 4-19.8Gy; RNI:NA	NA
			WB	178			
Qi W.X. et al./2020	Retrospective study	NA	WB+IMNI	58	N1	NA	6.67y
			WB	58			
Abdel-Rahman O. et al/2018	Retrospective study	NA	CW/WB+IMNI	136	N1	NA	6.33y
			CW/WB	676			
Kim H. et al/2017	Retrospective study	2006-2010	WB with SCN	271	N1	WB:50-50.4Gy; boost: 10-16Gy; RNI: 45-50.4Gy	6.08y
			WB alone	271			
ALTTO/2017	Retrospective study	NA	CW/WB+ mixed RNI*	878	N+ HER-2 positive IBC	Median RNI dose: 49-50Gy	4.5y
			CW/WB	786			
Aleknavicius E. et al/2014	Retrospective study	1987-1997	CW+SCN±AXL+IMN	165	pT1-2N0-1M0 IBC	CW: 50Gy/25Fx SCN: 40-44Gy/20-22Fx	8.5y
			CW+SCN±AXL	268			
Courdi A. et al/2013	Retrospective study	1975-2008	CW/WB+ mixed RNI*	406	N negative	NA	12.8y
			CW/WB	1042			
Chen X. et al/2013	Retrospective study	2000-2007	CW/WB+ mixed RNI*	93	T1-2N1 triple negative breast cancer	CW:46-50Gy/23-25FxRNI: 46-50Gy/23-25Fx	5.4y
			No RT	460			
Chang J.S. et a,/2013	Retrospective study	1994-2002	WB/CW+SCN±AXL+IMN	197	Stage II-III	CW/WB: 50.4Gy/28FxRNI: 50.4GY/28Fx	12.4y
			WB/CW+SCN±AXL	199			
Olson R. A. et al/2012	Retrospective study	2001-2006	WB/CW+SCN±AXL+IMN	529	N+ or T3/4N0	CW/WB: 42.5Gy/16FxRNI: 40GY/16Fx	6.2y
			WB/CW+SCN±AXL	779			
Truong P.T. et al/2009	Retrospective study	1989-1999	CW+ mixed RNI*	458	T1-2N1M0 IBC	WB: 40-50Gy/15-25Fx	8.6y
			No RT	5230		RNI:NA	

NA, not available; WB, whole breast; CW, chest wall; IBC, invasive breast cancer; RNI, regional node irradiation; IMN, internal mammary node; SCN, supraclavicular lymph node; AXL, axillary lymph node.

*Mixed RNI: the radiation volume of RNI is mixed including RNI+SCN and/or RNI+IMNI and/or RNI+SCN+IMNI±AXL.

### Quality of included studies

For the five prospective randomized studies (5, 15–17, 27), all of them were open-label randomized controlled trial, thus had a Jadad score of 3. For the remaining 14 retrospective trials and 1 non-randomized trial, the quality was evaluated according to the NOS table. All 15 studies had good quality, with a ≥6 (**Supplementary Table 1**).

### Effect of different RNI volumes on outcomes of BC

**Figure 2:**
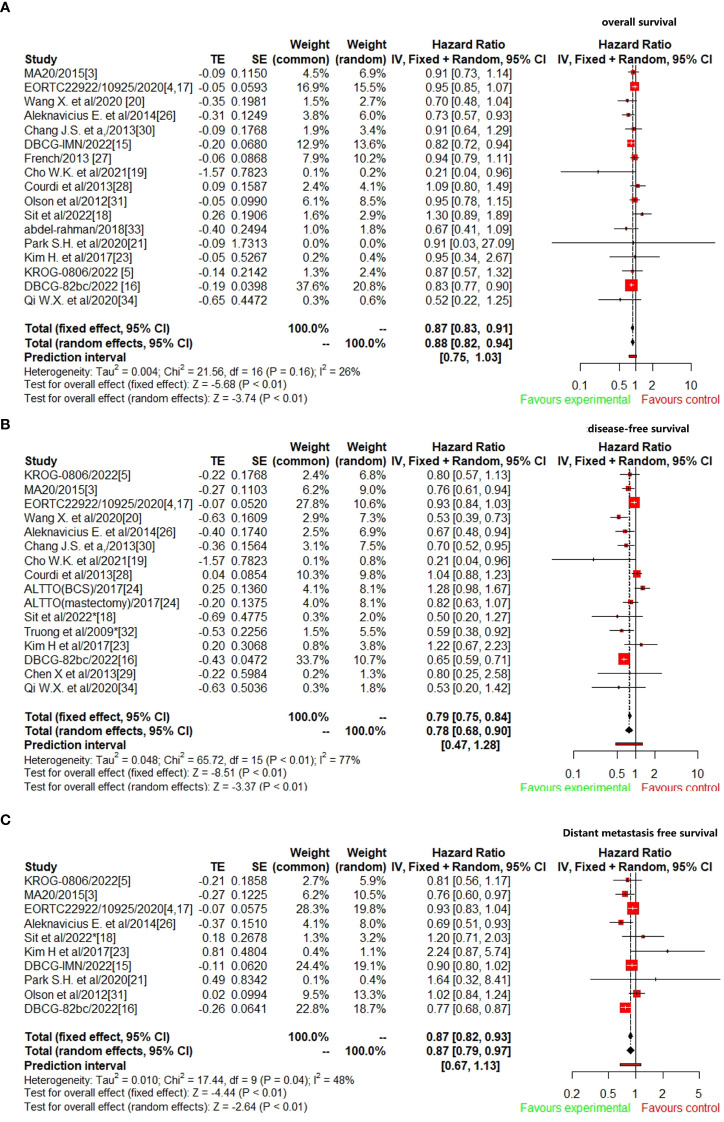
Comparison survival outcomes of RNI with IMNI vs. RNI without IMNI in early-stage BC. **(A)** OS; **(B)** DFS; **(C)** DMFS.

A total of 17 studies (3–5, 15–21, 26–28, 30, 31, 33, 34) reported OS data were included to analyze the efficacy of CW/WB+RNI with IMNI vs. without IMNI in BC patients. The pooled hazard ratio for OS demonstrated that CW/WB+RNI with IMNI in BC patients significantly improved OS, giving HR of 0.87 (95%CI: 0.83–0.91, *p*<0.01, **Figure 2A**). There was no significant heterogeneity between studies (*I* ^2^=26%, *p*=0.16), and the pooled HR for OS was performed by using fixed-effects model. As for DFS, 16 studies were included for analysis (5, 15–20, 23, 24, 26, 28–30, 32, 34). The pooled result showed that CW/WB+RNI with IMNI in BC patients significantly improved DFS with an HR of 0.78 (95%CI: 0.68–0.90, *p*<0.01, **Figure 2B**). There was significant heterogeneity between studies (*I* ^2^=77%, *p*<0.01), and the pooled HR for DFS was performed by using random-effects model. A total of 10 studies included DMFS data for analysis (5, 15–18, 21, 23, 25, 26, 31), and the pooled results showed that CW/WB+RNI with IMNI in BC patients significantly improved DMFS giving HR of 0.87 (95%CI: 0.79–0.97, *p*<0.01, **Figure 2C**). There was significant heterogeneity between studies (*I* ^2^=48%, *p*=0.04), and the pooled HR for DMFS was performed by using random-effects model.

### Sub-group analysis according to different RNI volumes

**Figure 3:**
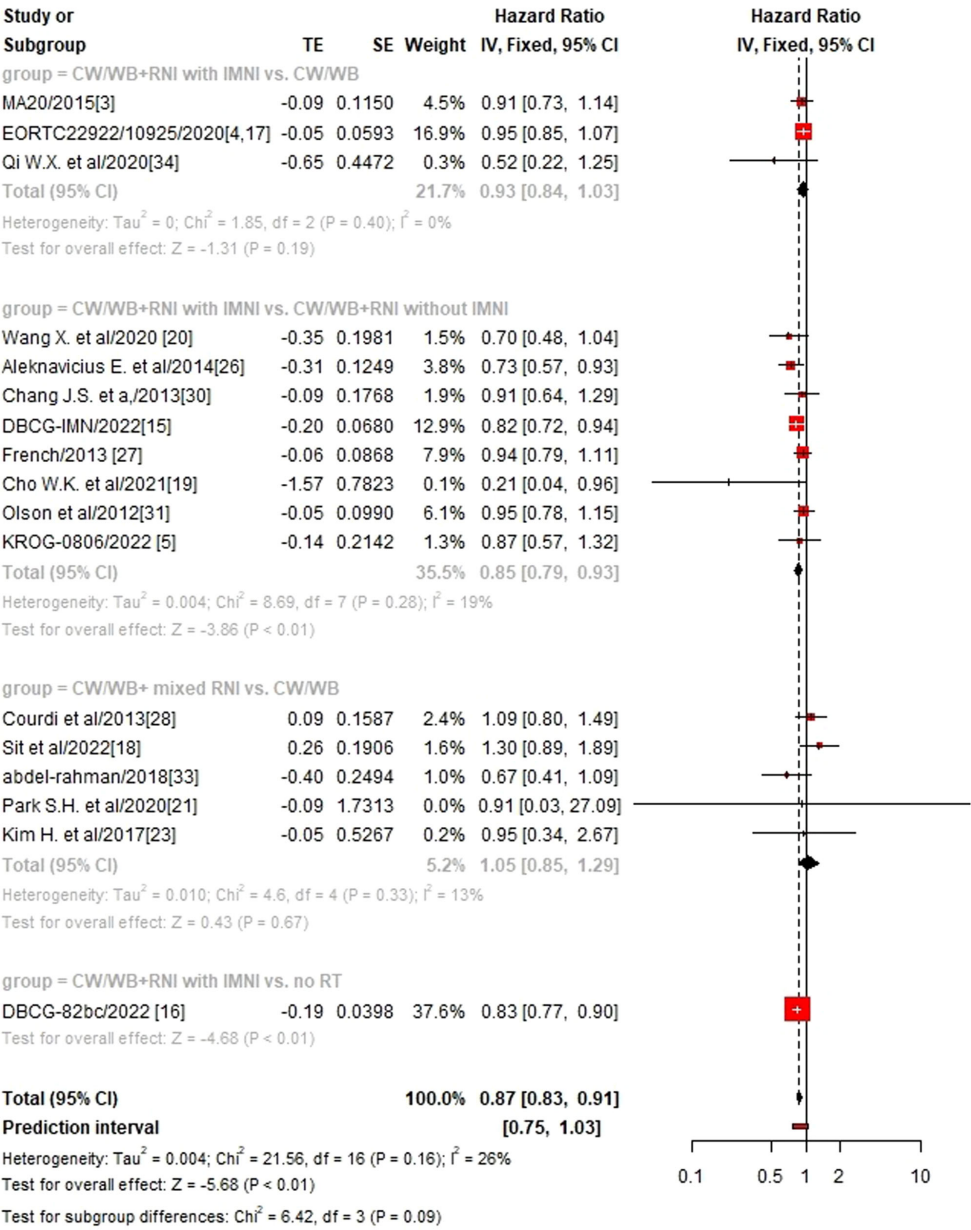
Sub-group analysis of OS according to RNI volume in early-stage BC.

We then performed sub-group analysis according to different RNI volumes and found that CW/WB+RNI with IMNI significantly improved OS (HR 0.85, 95%CI: 0.79–0.93, p<0.01, **Figure 3**), DFS (HR 0.65, 95%CI: 0.56–0.77, p<0.01, **Supplementary Figure 1**), and DMFS (HR 0.90, 95%CI: 0.82–0.98, p=0.02, **Supplementary Figure 2**) when compared to CW/WB+RNI without IMNI, while no significant difference could be found between CW/WB+RNI with IMNI vs. CW/WB in terms of OS (HR 0.94, 95%CI: 0.85–1.03, p=0.19) and DFS (HR 0.93, 95%CI: 0.86–1.00, p=0.06), but not for DMFS (HR 0.90, 95%CI: 0.81–0.99, p=0.04). Similarly, CW/WB+ mixed RNI did not significantly improve the OS (HR1.05, 95%CI: 0.85–1.29, p=0.67), DFS (HR 0.94, 95%CI: 0.80–1.11, p=0.48), and DMFS (HR 1.41, 95%CI: 0.91–2.19, p=0.13) of BC patients treated with CW/WB alone.

### Network meta-analysis

For quantitative synthesis within NMA, treatment approaches from eight studies were categorized into groups as follows: 1) CW/WB+RNI with IMNI-R (right-side breast cancer), 2) CW/WB+RNI with IMNI, 3) CW/WB+ RNI without IMNI, 4) CW/WB +mixed RNI, 5) SW/WB+SVC, 6) CW/WB alone, and 7) no radiotherapy (RT).

**Figure 4:**
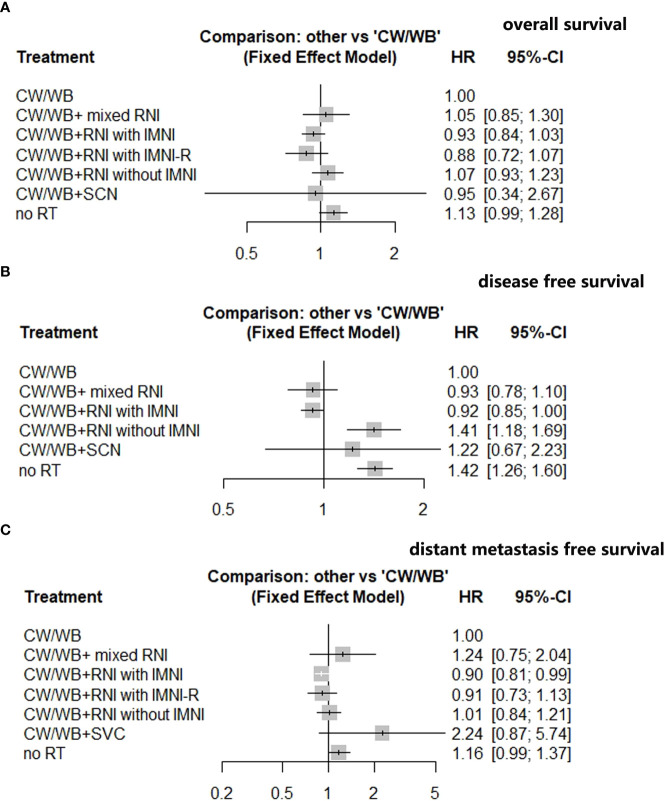
Network analysis of different RNI volume versus CW/WB alone early-stage BC. **(A)** OS; **(B)** DFS; **(C)** DMFS.

An NMA of seven different RNI radiotherapy regimens was conducted with regards to OS in BC patients (**Supplementary Figure 3A**). Compared to CW/WB, CW/WB+RNI with IMNI had a tendency to improve OS (HR 0.93, 95%CI: 0.84–1.03, **Figure 4A**), while no significant survival difference could be observed between CW/WB+RNI with IMNI-R and CW/WB (HR 0.88, 95%CI: 0.72–1.07, **Figure 4A**). Similarly, CW/WB+RNI without IMNI did not improve the OS when compared to CW/WB alone (HR 1.07, 95%CI: 0.93–1.23, **Figure 4A**). However, RT did not significantly decrease the OS when compared to CW/WB in BC patients (HR 1.13, 95%CI: 0.99–1.28). p-score for each treatment is shown in **Supplementary Table 2**. In the BC patients, CW/WB+RNI with IMNI-R had the highest p-score for OS followed by CW/WB+RNI with IMNI, indicating that it was a better treatment option for preventing breast cancer death based on treatment ranking according to p-score (**Supplementary Table 2**).

An NMA of six different maintenance therapy regimens was conducted with regards to DFS in BC patients (**Supplementary Figure 3B**). Compared to CW/WB, CW/WB+RNI with IMNI significantly improved DFS (HR 0.92, 95%CI: 0.85–1.00, **Figure 4B**), while CW/WB+RNI without IMNI (HR 1.41, 95%CI: 1.18–1.69, **Figure 4B**), CW/WB+ mixed RNI (0.93, 95%CI: 0.78–1.10, **Figure 4B**) or CW/WB+SVC (HR 1.22, 95%CI: 0.67–2.23, **Figure 4B**) was not significantly associated with a lower likelihood of disease progression. According to the p-score for each treatment, CW/WB+RNI with IMNI had the highest p-score (**Supplementary Table 3**).

An NMA of seven different RNI radiotherapy regimens was conducted with regards to DMFS in BC patients (**Supplementary Figure 3C**). Compared to CW/WB, CW/WB+RNI with IMNI significantly improved DMFS (HR 0.90, 95%CI: 0.81–0.99, **Figure 4C**), while CW/WB+RNI without IMNI-R (HR 0.91, 95%CI: 0.73–1.13, **Figure 4C**), CW/WB+RNI without IMNI (HR 1.01, 95%CI: 0.84–1.21), CW/WB+ mixed RNI (1.24, 95%CI: 0.75–2.04, **Figure 4C**), or CW/WB+SVC (HR 2.24, 95%CI: 0.87–5.74, **Figure 4C**) was not significantly associated with a lower likelihood of disease progression. According to the p-score for each treatment, CW/WB+RNI with IMNI had the highest p-score (p=0.8883, **Supplementary Table 4**). Dummy **Supplementary Table 5**, **6**

### Publication bias and sensitivity analysis

As shown in **Supplementary Figure 4**, there was no obvious asymmetry in modified funnel plots for OS, DFS, and DMFS, indicating the absence of significant publication bias. We also performed Egger’s tests to detect the publication bias, and no statistically significant publication bias was detected for OS (Egger’s test, p=0.74), DFS (Egger’s test, p=0.71), DMFS (Egger’s test, p=0.43). The sensitivity analysis (**Supplementary Figure 5**) was performed to test the stability of our findings. The results suggested that the effects of CW/WB+RNI with IMNI or mixed RNI therapy on OS, DFS, and DMFS were reliable.

## Discussion

Over the past decades, primary breast tumor resection followed by adjuvant radiation to the lymphatic drainage for both early-stage and locally advanced BC had become the standard treatment strategy. However, the treatment volume of RNI significantly varied among those published studies. Thus, there remained a matter of debate on which regional nodal volume should be irradiated for BC patients in the era of effective systematic therapies (35). Prior to the present study, Budach et al. performed two meta-analyses to define the optimal RNI volume based on four prospective randomized trials, and the pooled results indicated a statistically significant improvement in overall survival for CW/WB+ comprehensive RNI when compared to CW/WB alone in stage I–III breast cancer (36, 37). In 2019, the Early Breast Cancer Trialists’ Collaborative Group conducted an updated meta-analysis to investigate the efficacy of regional nodal irradiation in early-stage BC patients according to treatment periods and found that RT to regional lymph nodes in older (1961–1978) studies increased the overall risk of death, while nodal RT in more recent (1989–2003) studies reduced breast cancer recurrence, breast cancer mortality, and overall mortality (38). Since the publication of these meta-analyses, more clinical trials had been performed in recent years, but the optimal treatment RNI strategy remained undetermined. We therefore performed this meta-analysis to assess the benefit of different RNI treatments in BC patients and compare the efficacy of different RNI regimens via network meta-analysis.

A total of 29,640 BC patients from 20 studies were included for analysis. In consistent with previous result (39), our pooled HR demonstrated that radiotherapy with IMNI in BC patients significantly improved OS, DFS, and DMFS when compared to RNI without IMNI (p<0.05). We then performed a sub-group analysis according to RNI volume and found that CW/WB+RNI with IMNI significantly improved OS (HR 0.85, p<0.01), DFS (HR 0.65, p<0.01), and DMFS (HR 0.90, p=0.02) when compared to CW/WB+RNI without IMNI. In contrast to the report of Budach et al. (36), no significant difference could be found between CW/WB+RNI with IMNI vs. CW/WB alone in terms of OS (p=0.19) and DFS (p=0.48), while a significantly decreased risk of developing distant metastasis could be observed in CW/WB+RNI with IMNI group when compared to CW/WB alone (p=0.04). Additionally, CW/WB+ mixed RNI did not improve OS, DFS, and DMFS when compared to CW/WB alone (p>0.05). We further explored the optimal RNI treatment strategy for BC patients by using network meta-analysis. Based on those published studies, CW/WB+RNI with IMNI remained a better treatment option for preventing BC progression and death, and CW/WB combined with comprehensive RNI could be recommended for BC patients after curative surgery.

IMNI would inevitably increase RT dose to the heart and lungs regardless of using modern radiation technology, which might be associated with an increased risk of developing RT-related toxicities (40). But in a large cohort study based on SEER database, Giordano et al. (41). found that the risk of death from ischemic heart disease associated with radiation for breast cancer had been substantially decreased over time. In a more recent meta-analysis of three randomized trials, Budach et al. found that IMNI was not associated with an excess of cardiac death or cardiac toxicity rate (36). Although there was a lack of formal guidelines on whether or not to treat IMN for BC patients, radiation oncologists in our institute had achieved a consensus that CW/WB+RNI with IMNI could be recommended for BC patients due to the low incidence of cardiac toxicities. Between June 2017 and August 2019, we performed a prospective randomized controlled trial to investigate the early cardiac event in pre-specified dose constraints for the heart vs. choice of radiation oncologists for breast cancer treated with postoperative intensity-modulated radiotherapy, which had been registered in ClinicalTrials.gov (NCT02942615). By 31 December 2019, 143 patients completed 1-year follow-up (77 in study group and 66 in control group). The Dmean of the heart was 374.9 ± 205.3cGy and 376.7 ± 204.7cGy in the study and control group, respectively (p=0.96). No clinical cardiac toxicity was observed. Subclinical cardiac events occurred in 29 patients in the study group and 29 in the control group (p=0.45) (42). Therefore, mean heart dose in BC treated with IMNI was low and did not increase the risk of developing early-stage cardiac toxicities. As for late cardiac toxicities, EORTC Trial 22922/10925 reported 15-year side effects after IMNI and showed that IMNI did not significantly increase the risk of developing cardiac fibrosis when compared to without IMNI group (1.1% vs. 1.9%, p=0.07) but not for any cardiac disease (9.4% vs. 11.1%, p=0.04). RT technique improvement would also impact the cardiac toxicity of RT. The Danish Breast Cancer Group performed a population-based study and found a higher risk of cardiac toxicity in left- vs. right-sided patients irradiated during the non-CT-based period, while no increased risk of coronary artery disease in left-sided versus right-sided patients was observed in the CT-based period (43). As a result, with the improvement of RT techniques, IMNI could be safely applied for BC patients, even in the left-side BC.

Another major concern was that BC was a heterogeneous disease, and the risk of developing recurrence significantly varied. For BC patients with multiple risk factors, including young age, large tumor size, lymph vascular invasion, medial/central tumor location, or high nuclear grade (44), the incidence of local regional recurrence could increase to 20%. Therefore, one size did not fit all, and not all BCs could benefit from comprehensive RNI. Based on our established clinical risk model for N1 breast cancer (45, 46), our group initiated a prospective randomized clinical trial to investigate whether a part of pN1 breast cancer patients could be safely omitted from IMNI by using a clinical-genomic model, and the trial was under recruiting(NCT04517266). The preliminary results of enrolled 75 patients showed that among clinically high-risk pN1 BC, which is defined as having at least two of the five clinical risk factors (age≤40, three positive LN(lymph node), T2 stage, grade 3, and Ki-67 index≥14%), 70% of them present with genomic high risk, and 30% present with genomic low risk, and whether those clinical high-risk but genomic low-risk BC patients could be omitted from IMNI still needs long-term follow-up of our research (47). Another potential method was performing lymphoscintigraphy by injecting radiotracer for axillary sentinel node biopsy and peritumor to identify high risk for IMN metastases. It had been reported that lymphoscintigraphy not only identifies axillary sentinel node biopsy but also depicts drainage to the internal mammary (IM) basin, present in approximately 20% of patients (48, 49). Internal mammary sentinel lymph node biopsy (IM-SLNB) was another way to clearly determine the status of IMN metastases, although routine performance of IM-SLNB in clinical axillary lymph node (ALN)-negative patients remained debated. Qiu et al. (50) conducted a prospective cohort study and found that IM-SLNB visualization rate was 71.9% among BC patients who received initial surgery and 33.1% among BC patients treated with neoadjuvant systemic therapy.

In the real-world practice, there is a severe heterogeneity issue of RNI administration. In the ALTTO trial, only a third of the pN1 patients (36.8%) received RNI, while 82.2% of patients with four or more positive lymph nodes were treated with RNI. Among patients treated with RNI, 60.9% of the patients RNI targeting only one regional node area, while only 3.9% received RNI targeting the three regional nodal areas (24). One possible explanation for this finding was that the ALTTO trial was conducted between 2007 and 2011, before the first publication of the MA.20 and EORTC 22922/10925 trials. The quality of the regional nodal plan was another issue that should caution radiation oncologists. Ling et al. performed a network to determine the compliance with regional nodal coverage, contouring quality, target coverage, and organ-at-risk dosimetric parameters and found that 18% of plans presented with unacceptable nodal contour quality and 15% of them had inadequate coverage (51). In the KROG-0806 trial, the individual case review demonstrated that overall protocol compliance, including IMNI, significantly varied, and only 59.0% of the prescribed dose was delivered to the IMNI group (52, 53).

Nevertheless, there were some limitations that needed to be concerned of. First, both prospective and retrospective studies were included in this meta-analysis. Although the quality of included studies was high, selection bias between groups could not be avoided despite the fact that we performed subgroup analysis according to RNI volumes that successfully reduced the heterogeneity to a low grade. Second, this study was conducted at the base trial level but not at the individual level. Therefore, we could not perform pooled analysis according to patient characteristics, such as nodal stage or tumor location, although it had been established that medial-located tumor is a well-known predictor for IMNI benefit. In addition, both early-stage and locally advanced BC were included in the present meta-analysis, which might be another source of heterogeneity. Finally, the present study included both old or modern systematic therapy, and advances in systematic treatment in the modern era might affect the benefit of RNI, which might be another source of heterogeneity.

## Conclusion

In conclusion, the present study demonstrated that RNI with IMNI yielded a significant survival advantage for BC patients. Subgroup analysis according to RNI volumes showed that CW/WB+RNI+IMNI significantly improved DFS and DMFS when compared to CW/WB+RNI without IMNI, but not for OS. NMA found that CW/WB+RNI+IMNI was the optimum RNI treatment strategy for BC patients that reduced mortality and disease progression. Additionally, further studies evaluating the impact of RNI volume on late cardiac and lung toxicities are strongly needed.

## Data availability statement

The original contributions presented in the study are included in the article/**Supplementary Material**. Further inquiries can be directed to the corresponding authors.

## Author contributions

Conceptualization: JC and W-XQ. Project administration: CX and LC. Methodology: W-XQ, GC, and JC. Data curation: JC and CX. Formal analysis: W-XQ. Manuscript preparation: W-XQ, CX, GC, JC, and LC. Final approval of manuscript: all authors.
