# Supplementary figures and images for: The optimal regional irradiation volume for breast cancer patients: A comprehensive systematic review and network meta-analysis of published studies
Source: Front Oncol. 2023 Jan 31;13:1081201. doi: 10.3389/fonc.2023.1081201 (PMC9927229; doi:10.3389/fonc.2023.1081201)

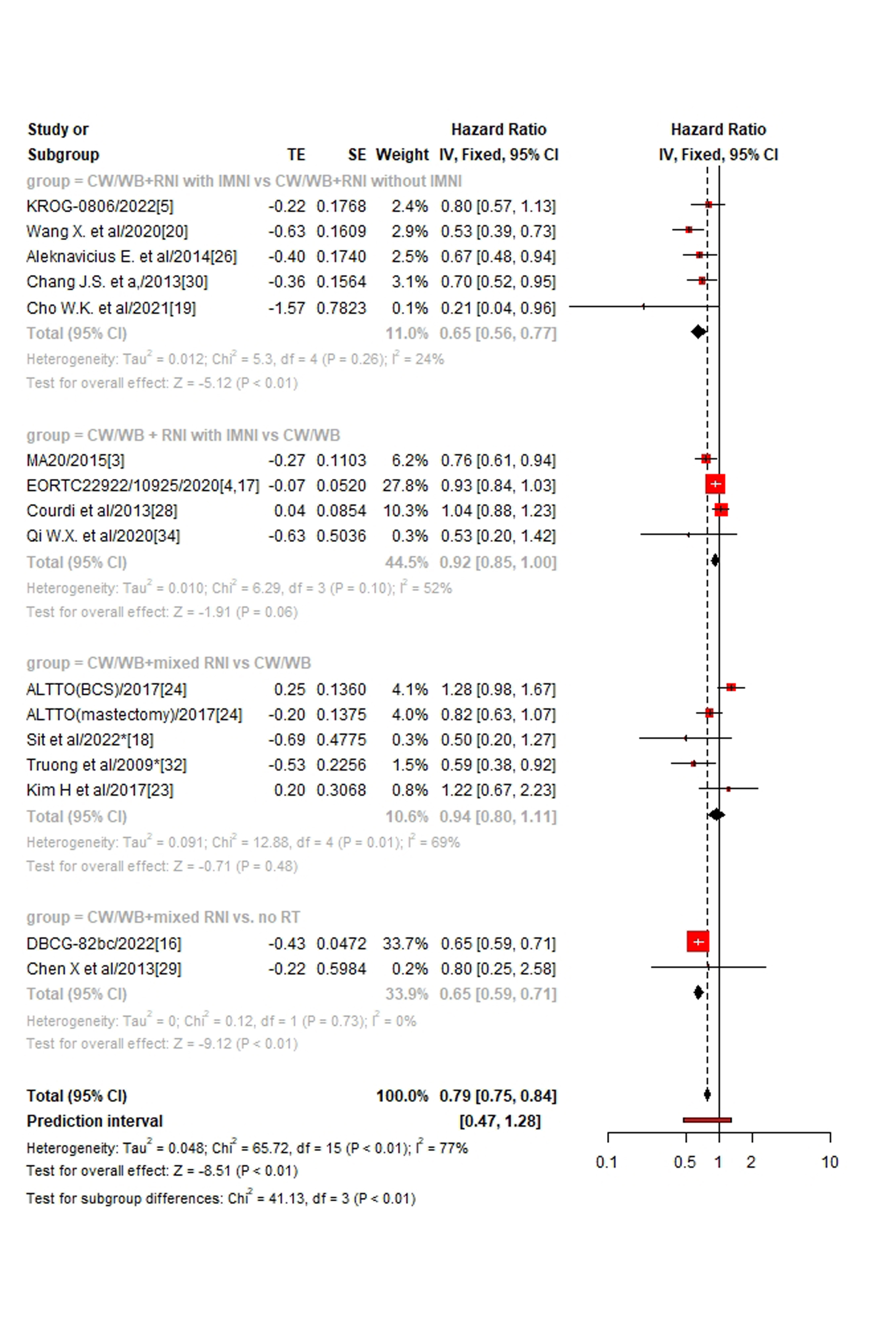

Supplement: Supplementary file 1 [file Image_1.jpeg]

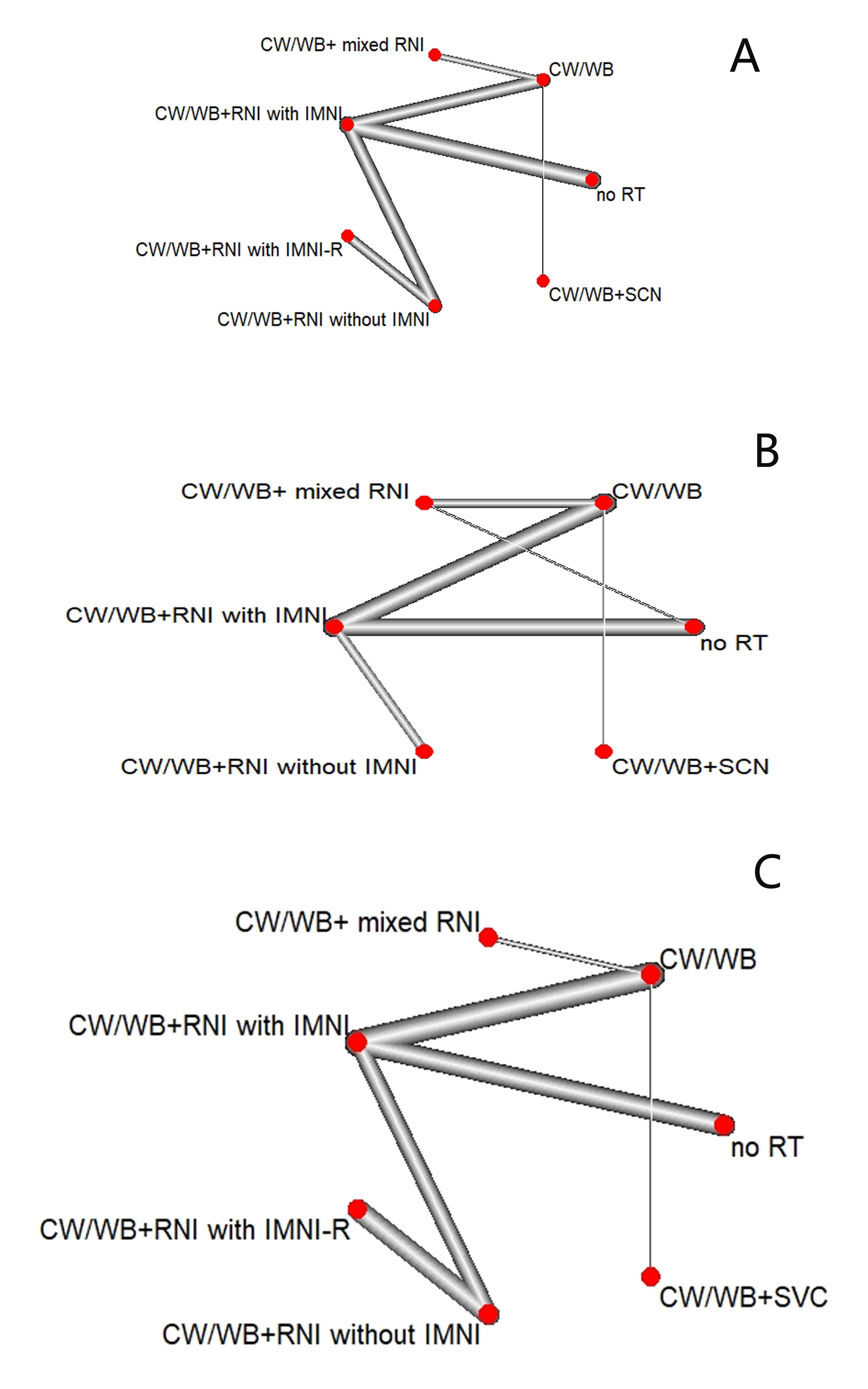

Supplement: Supplementary file 2 [file Image_2.jpeg]

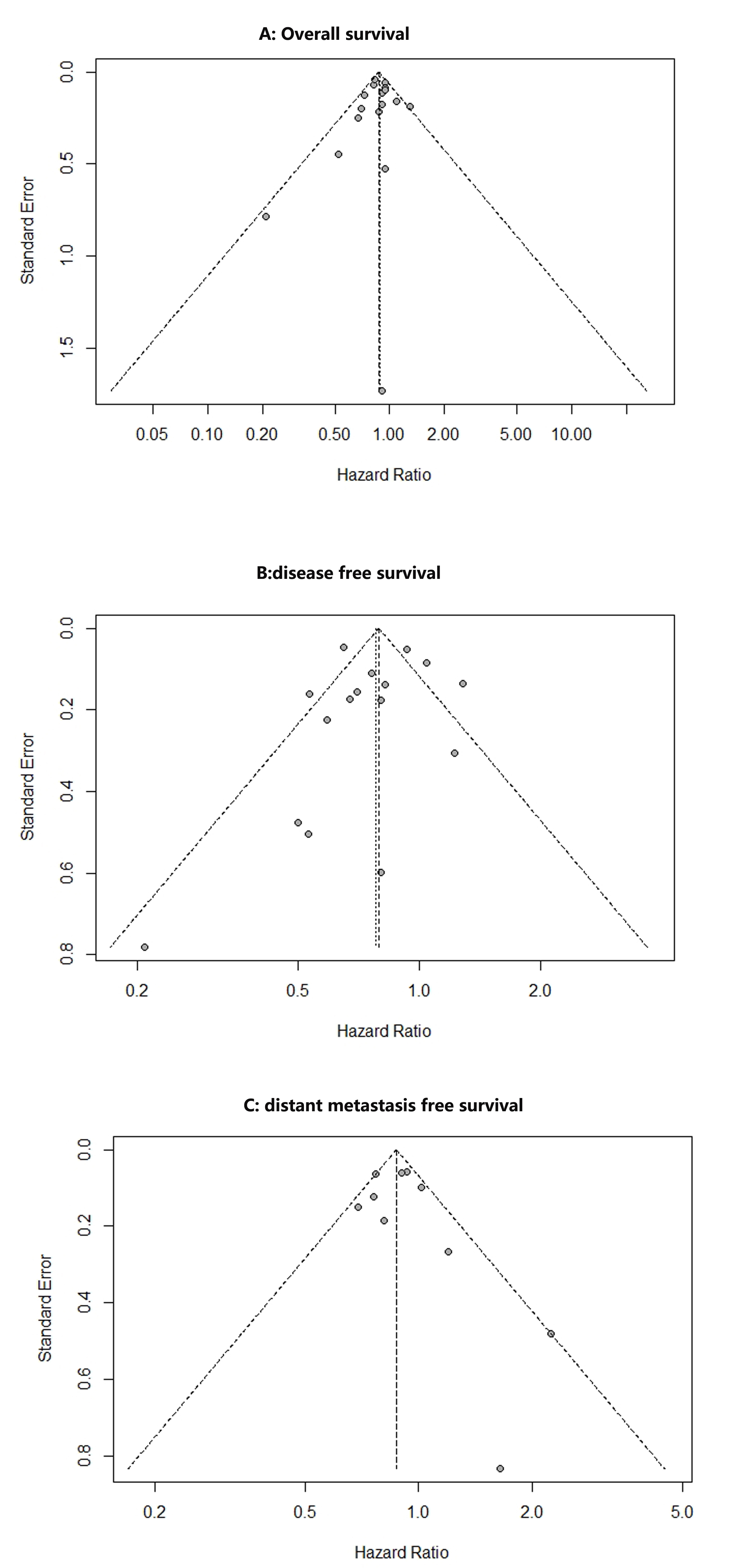

Supplement: Supplementary file 3 [file Image_3.jpeg]

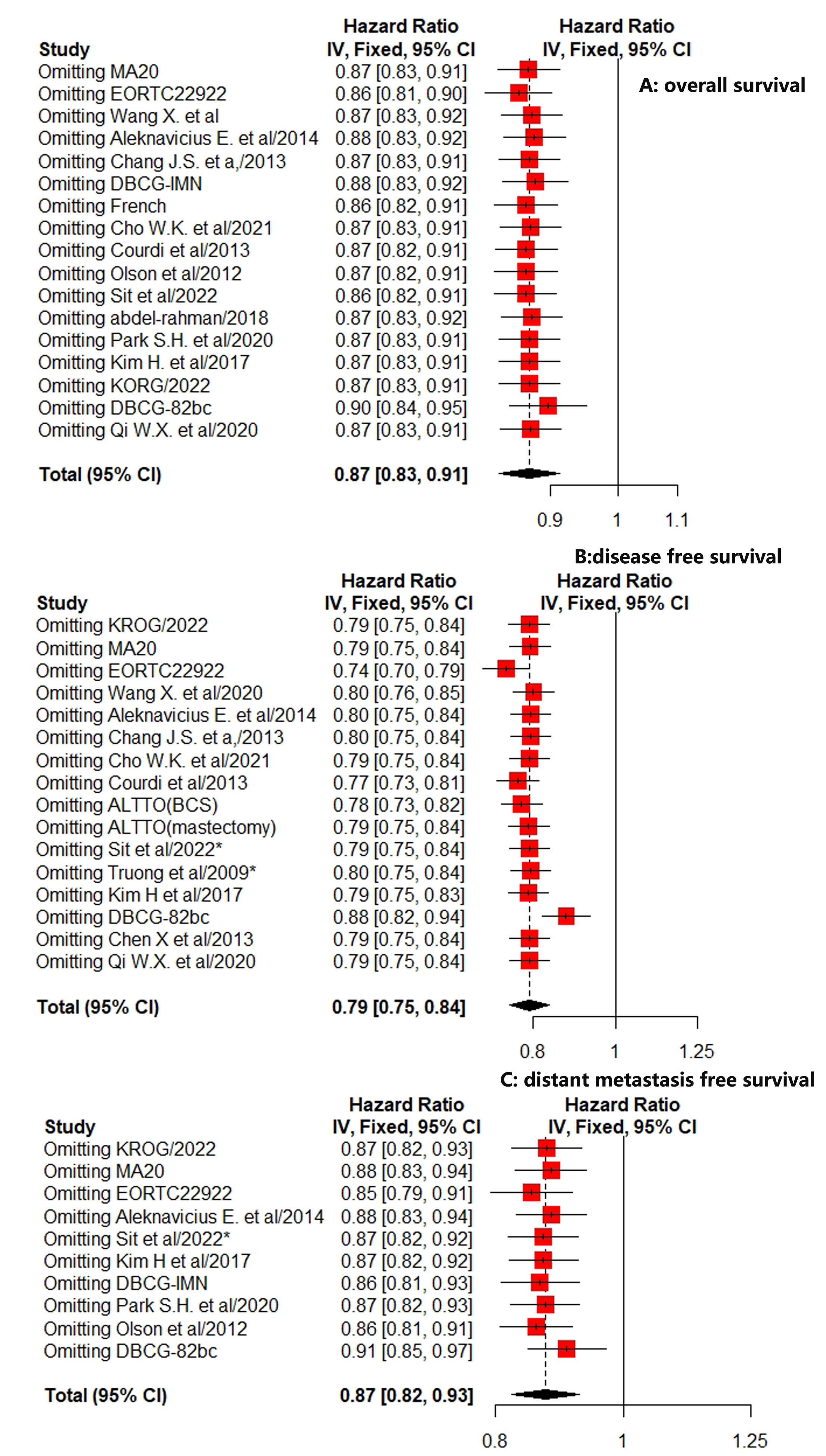

Supplement: Supplementary file 4 [file Image_4.jpeg]
