# Supplementary material for: The optimal regional irradiation volume for breast cancer patients: A comprehensive systematic review and network meta-analysis of published studies
Source: Front Oncol. 2023 Jan 31;13:1081201. doi: 10.3389/fonc.2023.1081201 (PMC9927229; doi:10.3389/fonc.2023.1081201)
Supplement: Supplementary file 5 [file Table_1.docx]

**Supplemental Table 1.** Results of quality assessment for included nonrandomized or retrospective studies using Newcastle-Ottawa Scale for cohort studies.

| **Studies** | **Selection** | | | | **Comparability** | | **Outcome** | | | **Total Score** |
| --- | --- | --- | --- | --- | --- | --- | --- | --- | --- | --- |
|  | **Representativeness of the Exposed Cohort** | **Selection of the Non-Exposed Cohort** | **Ascertainment of Exposure** | **Demonstration That Outcome of Interest Was Not Present at Start of Study** | **Comparability of Cohorts on the Basis of the Design or Analysis** | | **Assessment of Outcome** | **Median Follow-Up Greater Than 6 Months** | **Adequacy of Follow-Up of Cohorts** |  |
|  |  |  |  |  | **Age** | **Single Tumor Type or Adjusted model** |  |  |  |  |
| **Sit et al/2022** | ***** | * | * | * | ***** | ***** | * | * | * | 9 |
| **DBCG-IMN/2022** | * | * | * | * | ***** | * | * | * | * | 9 |
| **Wang X. et al/2020** | ***** | * | * | * |  | * | * | * | * | 8 |
| **Cho W.K. et al/2021** | ***** | * | * |  |  | * | * | * | * | 7 |
| **Park S.H. et al/2020** | * | * | * |  |  | * | * | * |  | 6 |
| **Qi W.X. et al./2020** | ***** | * | * |  |  | * | * | * | * | 7 |
| **Abdel-Rahman O. et al/2018** | * | * | * |  |  | * | * | ***** | * | 7 |
| **Kim H. et al/2017** | * | * | * | * |  | * | * | * |  | 7 |
| **ALTTO/2017** | * | * | * |  |  | * | * | * |  | 6 |
| **Aleknavicius E. et al/2014** | * | * | * | * | ***** | * | * | ***** | * | 9 |
| **Courdi A. et al/2013** | ***** | * | * | * | * | * | * | * | * | 9 |
| **Chen X. et al/2013** | ***** | * | * | * |  | ***** | * | * | * | 8 |
| **Chang J.S. et a,/2013** | ***** | * | * | * |  | * | * | * | * | 8 |
| **Olson R. A. et al/2012** | * | * | * | * |  | * | * | * |  | 7 |
| **Truong P.T. et al/2009** | * | * | * | * | * | * | * | * | * | 9 |
