# Supplementary material for: The optimal regional irradiation volume for breast cancer patients: A comprehensive systematic review and network meta-analysis of published studies
Source: Front Oncol. 2023 Jan 31;13:1081201. doi: 10.3389/fonc.2023.1081201 (PMC9927229; doi:10.3389/fonc.2023.1081201)
Supplement: Supplementary file 6 [file Table_2.docx]

**Supplemental table 2: the optimal adjuvant radiotherapy regimen for overall survival of early-stage breast cancer**

| **Adjuvant RT regimens** | **P-score** |
| --- | --- |
| CW/WB+RNI with IMNI-R | 0.8538 |
| CW/WB+RNI with IMNI | 0.7461 |
| CW/WB+SVC | 0.5422 |
| CW/WB | 0.5179 |
| CW/WB +mixed RNI | 0.3794 |
| CW/WB+RNI without IMNI | 0.3060 |
| No RT | 0.1545 |
