# Supplementary material for: The optimal regional irradiation volume for breast cancer patients: A comprehensive systematic review and network meta-analysis of published studies
Source: Front Oncol. 2023 Jan 31;13:1081201. doi: 10.3389/fonc.2023.1081201 (PMC9927229; doi:10.3389/fonc.2023.1081201)
Supplement: Supplementary file 7 [file Table_3.docx]

**Supplemental table 3: the optimal adjuvant radiotherapy regimen for disease free survival of early stage breast cancer**

| **Adjuvant RT regimens** | **P-score** |
| --- | --- |
| CW/WB+RNI with IMNI | 0.8605 |
| CW/WB+ mixed RNI | 0.8207 |
| CW/WB | 0.5914 |
| CW/WB+SVC | 0.3994 |
| CW/WB+RNI without IMNI | 0.1702 |
| no RT | 0.1578 |
