# Supplementary material for: The optimal regional irradiation volume for breast cancer patients: A comprehensive systematic review and network meta-analysis of published studies
Source: Front Oncol. 2023 Jan 31;13:1081201. doi: 10.3389/fonc.2023.1081201 (PMC9927229; doi:10.3389/fonc.2023.1081201)
Supplement: Supplementary file 10 [file Table_6.docx]

Supplemental table 6: detailed search strategy

| Database | PICO | Search strategy |
| --- | --- | --- |
| PubMed | P | breast cancer OR breast tumor OR breast tumour OR breast neoplasms [MeSH Terms] |
|  | I, C | Adjuvant radiotherapy [MeSH Terms] OR conformal radiotherapy [MeSH Terms] OR irradiation OR external beam radiotherapy OR radiation |
|  | S | randomized controlled trial [pt] OR controlled clinical trial [pt] OR randomized [tiab] OR placebo [tiab] OR drug therapy [sh] OR randomly [tiab] OR trial [tiab] OR groups [tiab]) NOT (animals [mh] NOT humans [mh] |
| EMBASE | P |  |
|  | I, C |  |
|  | S |  |
|  |  |  |
